# Effects of Physical Practice and Imagery Practice on Bilateral Transfer in Learning a Sequential Tapping Task

**DOI:** 10.1371/journal.pone.0152228

**Published:** 2016-04-06

**Authors:** William M. Land, Binya Liu, Alberto Cordova, Ming Fang, Yufei Huang, Wan X. Yao

**Affiliations:** 1 Department of Kinesiology, Health, & Nutrition, University of Texas at San Antonio, San Antonio, Texas, United States of America; 2 Department of Electrical and Computer Engineering, University of Texas at San Antonio, San Antonio, Texas, United States of America; 3 College of Physical Education, Yangzhou University, Yangzhou, China; University Zurich, SWITZERLAND

## Abstract

Recent research on bilateral transfer suggests that imagery training can facilitate the transfer of motor skill from a trained limb to that of an untrained limb above and beyond that of physical practice. To further explore this effect, the present study examined the influence of practice duration and task difficulty on the extent to which imagery training and physical training influences bilateral transfer of a sequential key pressing task. In experiment 1, participants trained on the key pressing task using their non-dominant arm under one of three conditions (physical practice, imagery practice, and no practice). In a subsequent bilateral transfer test, participants performed the sequential task using their untrained dominant arm in either an original order or mirror-ordered sequence. In experiment 2, the same procedures were followed as in experiment 1 except that participants trained with their dominant arm and performed the bilateral transfer task with their non-dominant arm. Results indicated that with extended practice beyond what has been employed in previous studies, physical practice is more effective at facilitating bilateral transfer compared to training with imagery. Interestingly, significant bilateral transfer was only observed for transfer from the non-dominant to the dominant arm with no differences observed between performing the task in an original or mirror ordered sequence. Overall, these findings suggest that imagery training may benefit bilateral transfer primarily at the initial stages of learning, but with extended training, physical practice leads to larger influences on transfer.

## Introduction

Bilateral transfer, also referred to as intermanual transfer or cross-education, is a phenomenon whereby experience obtained by a trained limb has an impact on the untrained contralateral homologous limb. To this extent, the training conducted with one limb can have a positive effect on the performance of the same task while using the untrained limb. The phenomenon of skill transfer from the trained limb to the untrained homologous limb was first studied and reported over one hundred years ago [1, 2]. Since then, studies in the field have been aimed at examining the direction of bilateral transfer [3, 4], exploring the influence of task characteristics on bilateral transfer [5, 6], and applying bilateral transfer to enhancing the rehabilitation of stroke survivors [7, 8]. Although the effect of physical practice on bilateral transfer has been well documented, the effect of imagery practice with a trained limb on the untrained limb has received less attention. However, such insight could prove important given the effectiveness of imagery in facilitating motor skill learning [9, 10], increasing muscle strength [11, 12], enhancing the development of cognitive skill representations [13], and most importantly its potential to be an effective therapeutic technique for the rehabilitation of patients with motor impairments, such as stroke survivors.

Recently, work has begun to illuminate the influence of imagery on bilateral transfer. For example, two studies [14, 15] compared the effects of physical practice and imagery practice on bilateral transfer. Both studies indicated that along with physical practice, imagery practice could also contribute to significant bilateral transfer. Interestingly, the study by Amemiya, Ishizu [14], which required learning a sequential tapping task within a short training period (~5 min), showed a strong and significant advantage for imagery practice over physical practice in performances with both the trained limb (i.e., retention test) and untrained limb on a mirror-order test (i.e., bilateral transfer). This finding is consistent with previous studies [9, 16] that have reported an advantage for imagery practice over physical practice for tasks characterized by a high degree of complexity and cognitive demands.

When complexity and cognitive demands of a task are high, then physical practice may lead to retroactive interference, which could be detrimental to the transfer of a learned skill from a trained limb to the opposite untrained limb [16]. Specifically, Saltzman [17] suggests that physical practice may cause interference due to the interactive effects between the “effector level” and “task level”. The effector level refers to limbs involved in performing the task and the task level refers to the central nervous system in planning the task. In comparison, imagery practice only involves activities on a “task level.” In other words, the imagery practice only “thinks” how to perform the task. Therefore, it leads to less interference, which results in superior learning compared to physical practice with respect to bilateral transfer.

Another important observation associated with imagery practice is that a positive effect of imagery is normally found when training duration is less than 20 minutes [9]. Driskell, Copper [9] revealed that the longer the imagery practice, the weaker its effect. This might be due to the fact that participants in extended physical practice constantly receive both intrinsic and extrinsic feedback regarding their performance and its outcome, which is not the case for the participants in imagery practice. The lack of feedback in the imagery practice may lower participants’ motivation and may result in boredom [9].

With respect to bilateral transfer, the findings of Amemiya, Ishizu [14] that imagery facilitated bilateral transfer beyond that of physical practice is consistent with these observations that imagery is more beneficial for tasks of high complexity and with short practice durations. Recall that Amemiya, Ishizu [14] utilized only a short practice duration (~5 min) and examined bilateral transfer on a task requiring a mirror-order sequence. Previous studies [18, 19] have shown that a mirror-order sequence is more cognitively difficult for transfer than an original-order sequence. Thus, it is not surprising that Amemiya, Ishizu [14] found an advantage for imagery over physical practice.

While, Amemiya, Ishizu [14] provided a first glimpse of the findings regarding an effect of imagery practice on bilateral transfer, it still remains unknown whether practice sessions of longer duration or utilizing tasks that are less complex and cognitively demanding (e.g., original order task) would eliminate or reduce the benefit of imagery compared to physical practice. That is to say, physical practice may be equal, or superior to, imagery practice in both retention and bilateral transfer learning on tasks of longer duration and less complexity (e.g., mirror-order versus original-order sequence). Thus, the main purpose of Experiment 1 was to examine the effects of motor imagery practice and physical practice over an extended training period on the inter-manual transfer from the non-dominant hand to the dominant hand across both mirror and original-order sequences.

## Experiment 1: Method

### Participants

Forty-five right handed (self-reported) college students participated in this study. The study was approved by the Institutional Review Board for the Protection of Human Subjects in Research at the University of Texas at San Antonio, and all participants signed a written consent form before participating in the study. All participants were free from current or past history of neurological impairment/dysfunction known to affect neuronal conduction.

### Task and Apparatus

**Fig 1:**
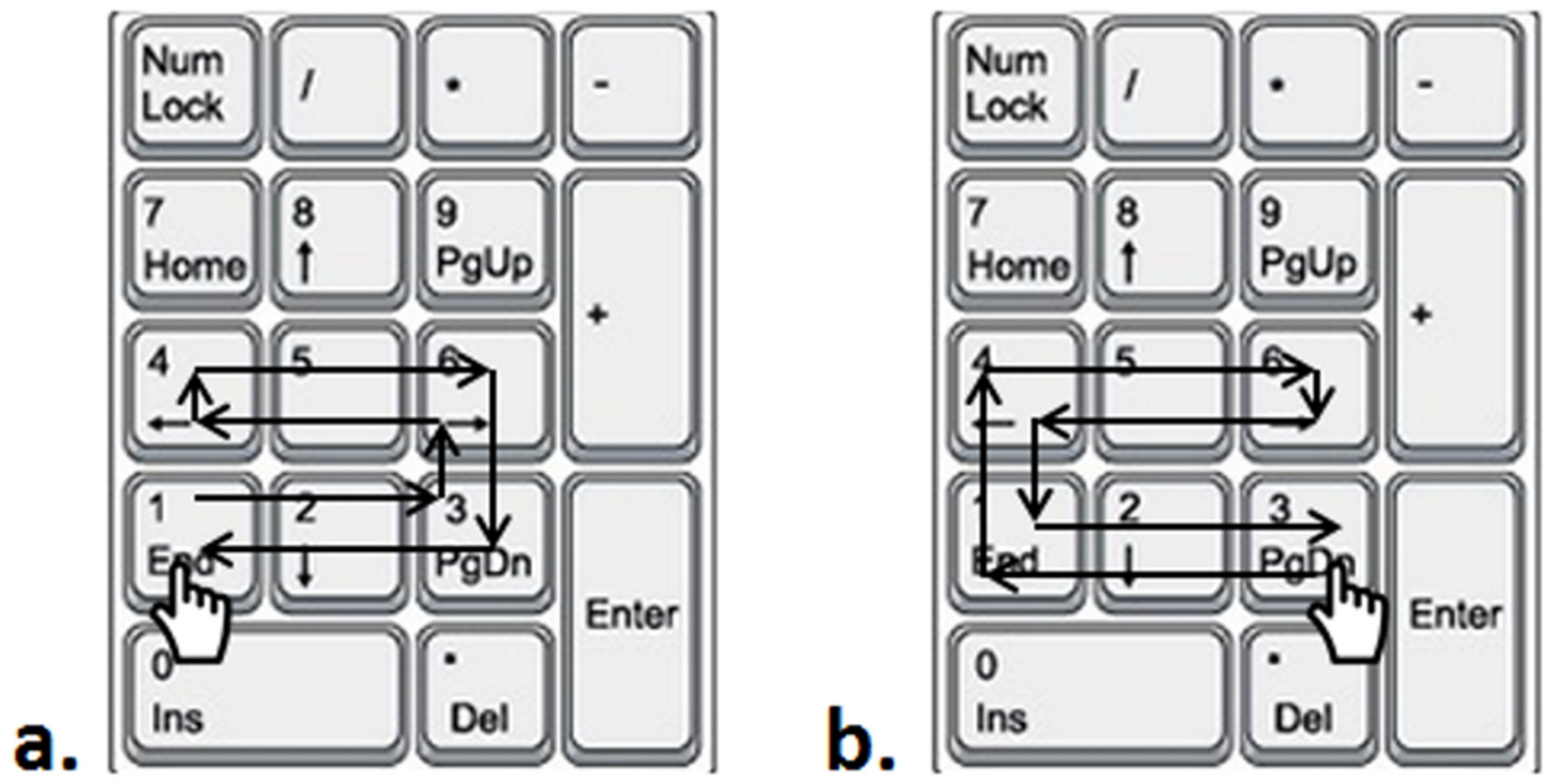
Key Pressing Task. Sequence of key presses for (a) original order and (b) mirror order key pressing task.

The participants were instructed to learn a sequential tapping task on a computer’s numerical keypad with their non-dominant hand (i.e., left hand). The task was the same as the one used by Amemiya, Ishizu [14], which required participants to tap keys in a fixed sequence, 1-2-3-6-5-4-4-5-6-3-2-1, on the numerical key pad by using the fingers on the left hand. The matching diagram of the keys and fingers were as follows: the participants started from left to right with the little finger, ring finger, and middle finger sequentially tapping on keypad #1, #2, and #3, respectively, and then followed from right to left with index finger, middle finger, and ring finger tapping on keypad #6, #5, and #4, respectively. Then, the participants were required to continue to tap the keypad numbers (i.e., 4, 5, 6, 1, 2, and 3) backward with the same fingers (see Fig 1a for original computer-key tapping orders).

For the transfer tests, the participants performed the task with their right hand in two different orders, original order and mirror order. With the original order, the participants started from left to right with the middle finger, ring finger, and little finger sequentially tapping on the keys #1, #2, and #3, respectively, followed with the ring finger, middle finger, and index finger tapping on the keys #6, #5, and #4 from right to left, respectively. Then, the participants tapped the keys (i.e., 4, 5, 6, 1, 2, and 3) backward with the same fingers (see Fig 1a). For the mirror-order test, the participants tapped the same keys with the same fingers as in the original-order transfer test but in a different order (i.e., 3, 2, 1, 4, 5, 6, 6, 5, 4, 3, 2, and 1) (see Fig 1b for mirror computer-key tapping orders).

Surface electromyography (EMG) activity of the left and right extensor digitorum communis (EDC) muscles during the practice phase was recorded with a MP150 Biopac system (Biopac systems, Inc. Santa Barbara, CA). The EMG signals were amplified (×1,000) and digitized (2,000 samples/s) by the MP 150 Data Acquisition System. The surface EMG recordings of each individual’s EDC muscles were taken for the purpose of checking if significant coactivity from the muscle on the non-trained hand occurred when the trained hand was performing.

### Procedures

#### Practice Phase

The participants in all groups (control, physical practice, & imagery practice) were informed and were shown the task by an experimenter. After the introduction of the task, the participants in the physical group performed 60 trials of the task with their left hand, which were divided into 6 blocks of 10 trials, with a 3-sec break between each trial and a 30-sec break between each block. Furthermore, they were encouraged to complete the task as quickly and as accurately as possible. The participants could visually observe their own fingers movements and were allowed to correct errors, if any. The total practice duration was approximately 40 min. The participants in the imagery group watched a video of the practice task performed by the left hand. The participants in this group were asked to imagine themselves doing the same task with the same side hand as shown in the video. Imagery training lasted approximately 40 min. Participants in the control group were required to read the fairy tale “Hansel and Gretel” during the practice phase.

#### Testing Phase

After a 5 min interval following the practice phase, participants performed a left-hand original-order test (e.g., a retention test). Participants then performed two transfer tests, an original-order test and a mirror-order test, using the untrained hand (i.e., right hand). Each test consisted of 10 trials and the order of the two transfer tests was pseudo counterbalanced.

### Data Acquisition and Analysis

During the practice phase, surface EMG data was recorded on both hands of the participants in the practice groups (physical practice and imagery practice groups). Movement time (MT) was calculated as the time of the first key press to the time of the last key press. Errors, if any, during the practice and test trials were also recorded. A video camera was used during the tests to record hand movement during the key pressing task. The recorded video was reviewed off line and acted as a second check for movement errors.

Error rates during the test phase were calculated by the number of error trials divided by the total number of practice trials. Any key presses not following the required sequence or using a wrong finger to tap was considered to be an error. The participants were all instructed to limit the error rate to or less than 5%.

The MT of the practice trials (i.e., the physical group) was analyzed with a one-way repeated-measure analysis of variance (ANOVA) (practice blocks). The MT of the test trials was analyzed with a 3 x 3 (Group x Test Type) mixed-model ANOVA with repeated-measures on the second factor. Statistical significance was set at *p* ≤ .05.

## Experiment 1: Results

### Error Rate

All participants maintained error rates at or below 5%. Thus, no further analyses of the error trials were performed.

### Muscle Activity during the Task

EMG records taken during the practice phase for the physical group were analyzed to examine if any significant muscle coactivations occurred on the untrained (right) EDC muscle when the trained (left) EDC exerted force. In addition, EMG records for the imagery group were also analyzed to see if any significant muscle activations occurred during practice. Results indicated no significant coactivation on the right EDC for either the physical group or imagery group. That is, the average value of the rectified surface EMG during the practice period (i.e., exerting forces) was within a range of the mean ±3 SD of the rectified surface EMG during the resting period (i.e., the baseline).

### MT of the Practice Blocks

**Fig 2:**
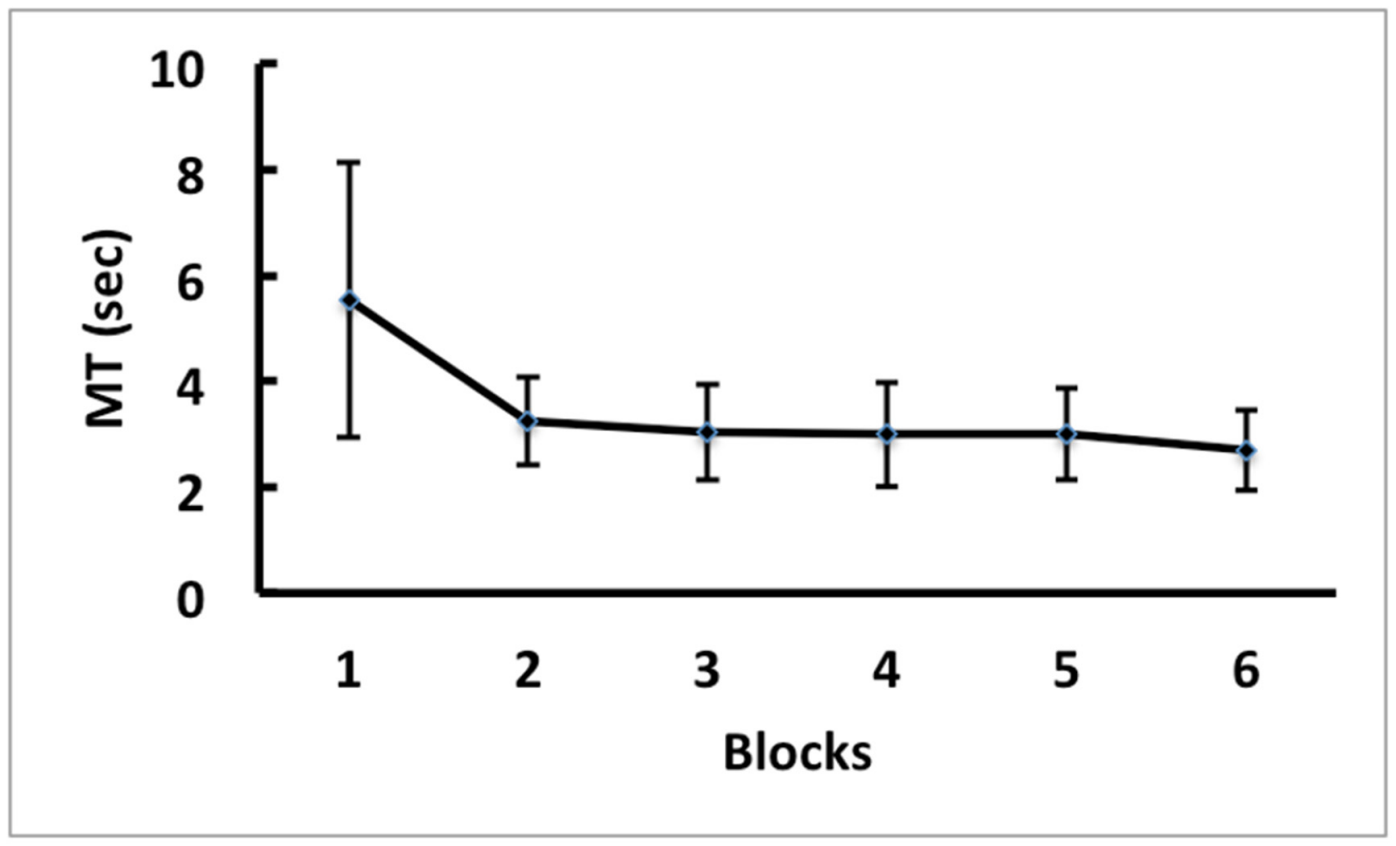
Practice Phase MT. Means and SDs of movement time (MT) of the practice trials in Experiment 1 for the physical practice group.

Fig 2 presents the means and standard deviations (SDs) of MT for the physical practice group during the practice phase. A one-way repeated-measure ANOVA showed a significant effect for block, *F*(5,70) = 17.88, *p* < .01. Post-hoc Paired Samples t-tests indicated that the first two blocks were significantly slower than the last four blocks. There was no significant difference in any other pair comparison.

### MT of the Posttest

**Fig 3:**
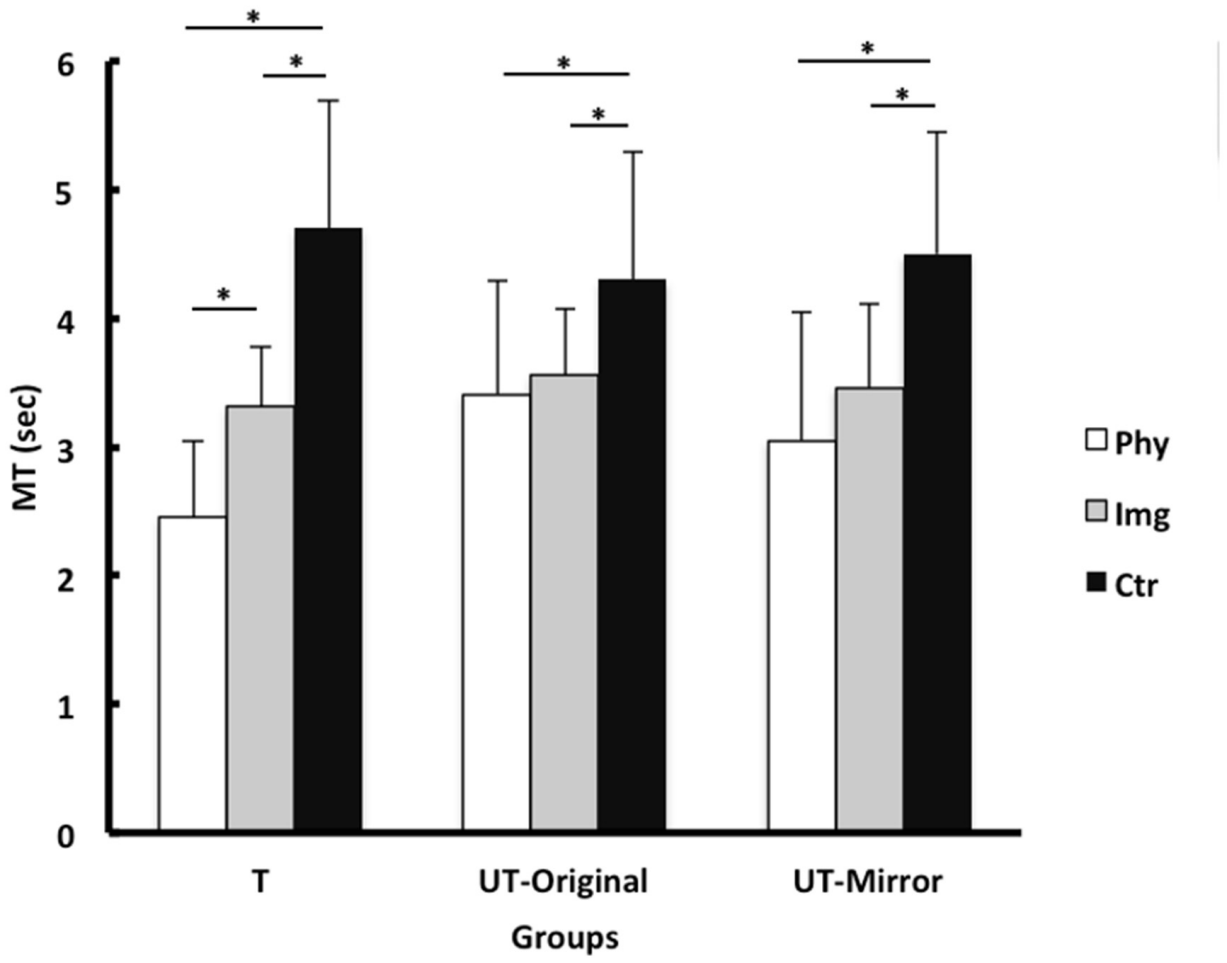
Posttest MT. Means and SDs of movement time (MT) of the posttest trials across each training condition for the trained hand test (T), untrained hand mirror ordered test (UT-Mirror), and untrained hand original order test (UT-Original) conditions in Experiment 1.

Fig 3 presents the means and SDs of MT for the trials during the test phase. The 3 x 3 (Group x Test Type) ANOVA with repeated measures on the second factor indicated a significant main effects for both Group and Test Type, *F*(2, 42) = 19.50, *p* < .001 and *F*(2, 84) = 3.46, *p* = .044, respectively. More importantly, the interaction effect of the two factors was also significant, *F*(4, 84) = 3.77, *p* < .01.

Due to the interaction effect, three separate one-way ANOVAs were conducted on each of the three posttests (i.e., trained-hand test, untrained-hand original-order test, and untrained-hand mirror-order test). The ANOVA for the trained-hand test indicated a significant group effect, *F*(2, 42) = 36.73, *p* < .001. A Bonferroni post hoc test indicated that the physical group was significantly faster (*M* = 2.45s, *SD* = .61) than both the imagery (*M* = 3.31s, *SD* = .47) and the control group (*M* = 4.70s, *SD* = .99). Furthermore, the imagery group was significantly faster than the control group. The ANOVA for the untrained-hand original-order test produced a significant group effect, *F*(2, 42) = 4.77, *p*< .05. A Bonferroni post hoc test indicated that the physical practice (*M* = 3.42s, *SD* = .89) and imagery groups (*M* = 3.57s, *SD* = .52) were both significantly faster than the control group (*M* = 4.38s, *SD* = 1.03). No significant difference in MT existed between the imagery and physical practice group. Finally, as to the untrained-hand mirror-order test, the ANOVA revealed a significant group effect, *F*(2, 42) = 8.57, *p*< .001. A Bonferroni post hoc test indicated that both physical practice (*M* = 3.05s, *SD* = 1.01) and imagery groups (*M* = 3.47s, *SD* = .65) were significantly better than the control group (*M* = 4.54s, *SD* = .95) with no significant difference between the physical group and imagery group.

## Experiment 1: Discussion

The first experiment examined the relative effects of physical practice and imagery practice over an extended practice duration on the learning of a sequential tapping task using both a trained non-dominant limb and untrained dominant limb (i.e., bilateral transfer with mirror- and original-order sequences).

With respect to performance during the test phase using the trained non-dominant limb, the results of Experiment 1 supported the assumption that increasing the duration of the practice session would reduce or eliminate the advantage of imagery over physical practice. That is, the physical group significantly out-performed the imagery group in the posttest with the trained hand, which is contradictory to the previous findings of Amemiya, Ishizu [14], which utilized only a very short practice duration (~5 min). This result is not surprising because previous research (e.g., 9) has shown that, with relatively longer practice durations (e.g., > 20 min), physical practice is more likely to outperform imagery practice when using a trained limb. As argued by Driskell, Copper (9), the availability of performance feedback during physical practice would provide essential information that could be used to enhance motor learning while also helping to maintain high levels of motivation. In contrast, the lack of such feedback during imagery training provides no direction for the learner to make corrections and improvements for future trials. Furthermore, this lack of feedback may also lead to lower levels of motivation in the participant.

Although our results suggest that an extended practice duration can reduce or eliminate interference caused by physical practice, they do not provide insight into how this reduction or elimination happened. One possibility is that with continued practice, there are fewer demands on cognitive resources directed to the task level (e.g., figuring out the sequence of the movement) and effector level (attention directed to movement kinematics) [9]. As a result, interference diminishes and there are fewer compromises to cognitive resources. However, a goal of future research should be to determine how such reduction of interference occurs.

Although the physical practice group outperformed the imagery group with the trained limb, surprisingly, the physical group did not outperform the imagery group on both mirror- and original-order bilateral transfers tests using the untrained limb. This result seems to be inconsistent with Driskell, Copper’s [9] prediction. According to Driskell, Copper [9], extended practice duration would enhance motor skill learning more for a physical practice group than an imagery practice group because the physical group could take advantage of the feedback from trial-and-error during learning. However, we assume that the feedback received from trial-and-error learning may primarily benefit the trained limb, with little to no impact on the untrained limb. Although our results indirectly support this assumption, future research should be done to address whether the advantage of trial-and-error feedback in enhancing motor skill learning is effector specific (e.g., muscles, joints, and limbs).

Frank, Land [13] suggest that the more cognitive a task is, the more it might benefit from imagery practice, as imagery is suggested to aid in the structuring of the memorial information that serves to guide movement execution. Previous studies [18, 19] suggest that a mirror-order sequence has a higher cognitive demand during transfer than an original-order sequence. Considering the aforementioned findings, it would be logical to assume that the imagery group should be better with respect to transfer requiring a mirror-order sequence than the physical group. However, this assumption was not supported by our results, because the two groups showed no significant difference in either transfer tests (i.e., mirror- and original-order sequence). It is likely that the extended practice duration allowed both physical and imagery practice group to have reached the same level of gains in terms of structuring the memorial information at the cognitive level, which serves to guide movement execution. However, this proposal is premature and needs to be confirmed in future studies.

Compared to the control group, both the physical and imagery practice groups showed significant positive bilateral transfer from the non-dominant limb to the dominant limb. This finding is counter to the findings by Lohse, Godde’s [15] in which they found significant transfer of production time as a result of imagery practice from the dominant hand to non-dominant hand direction only, and not in the opposite direction. The discrepancy between the two studies might be due to the differences in the tasks used by the studies. A computer keyboard tapping task used in our experiment is considered to be more of a visuomotor skill due to its high spatial constraints whereas the hand writing task, used in Lohse, Godde’s [15] study, would be considered to be more of a dynamical skill in terms of self-paced movement trajectories. According to previous studies, visuomotor skill favors the transfer from the non-dominant hand to the dominant hand [20], whereas dynamical skills favors the transfer from dominant hand to the non-dominant hand [21]. Therefore, the main purpose of the second experiment was to examine if the transfer effects observed in Experiment 1 with keyboard tapping is directionally sensitive.

## Experiment 2: Method

### Participants

Forty-five right handed (self-reported) college students who did not participate in the first experiment served as the participants for experiment 2. All participants were free from current or past history of neurological impairment/dysfunction known to affect neuronal conduction.

### Task, Apparatus, Procedures

The task, apparatus, and experimental procedures were the same as in the first experiment except that the participants learned the task with their dominant hand (right hand). For the transfer tests, the participants performed the sequential task with their non-dominant, left hand in two different orders, namely original order and mirror order.

Again, surface electromyography (EMG) activity of the left and right extensor digitorum communis (EDC) muscles during the practice phase was recorded with a MP150 Biopac system (Biopac systems, Inc. Santa Barbara, CA). The EMG signals were amplified (×1,000) and digitized (2,000 samples/s) by the MP 150 Data Acquisition System. The surface EMG recordings of each individual’s EDC muscles were taken for the purpose of checking if significant coactivity from the muscle on the non-trained hand occurred when the trained hand was performing.

### Data Acquisition and Analysis

The data was collected and analyzed in the way same as in Experiment 1. That is, the MT of the practice trials (i.e., the physical group) was analyzed with a one-way repeated-measure analysis of variance (ANOVA) (practice blocks). The MT of the posttest trials was analyzed with a 3 x 3 two-way (Group x Test Type) ANOVA with repeated-measures on the second factor. All statistical significance was set at *p* ≤ 0.05.

## Experiment 2: Results

### Muscle Activity during the Task

EMG records taken during the practice phase for the physical group were analyzed to determine if any significant muscle coactivations occurred on the untrained (right) EDC muscle when the trained (left) EDC exerted force. In addition, EMG records for the imagery group were also analyzed to see if any significant muscle activations occurred during practice. As in Experiment 1, the results of the EMG records in Experiment 2 did not show any significant coactivation on the right EDC for the physical nor imagery group.

### MT of the Practice Blocks

**Fig 4:**
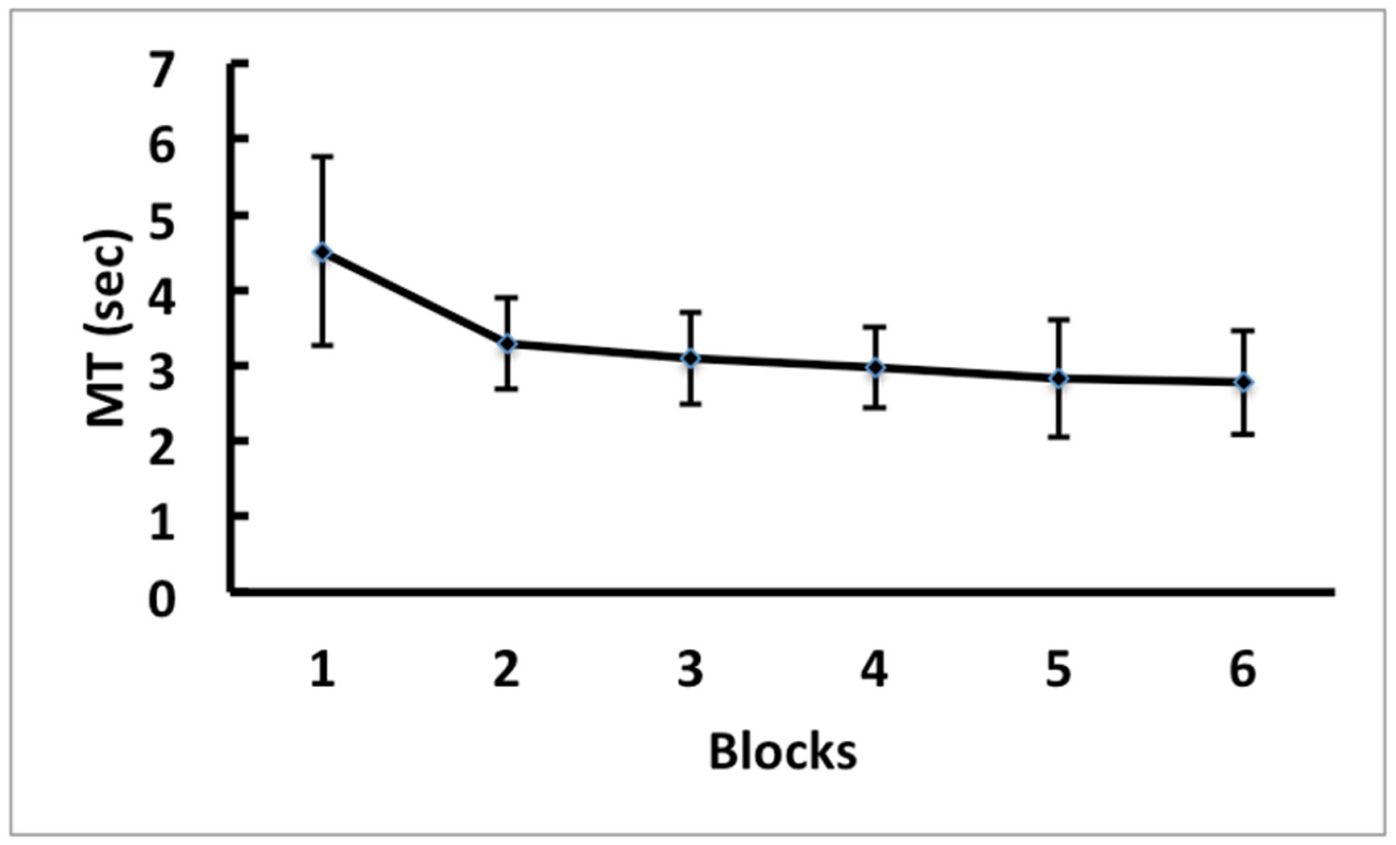
Practice Phase MT. Means and SDs of movement time (MT) of the practice trials in Experiment 2 for the physical practice group.

Fig 4 presents the means and standard deviations (SDs) of MT during the practice phase for the physical practice group. The one-way repeated-measure ANOVA indicated a significant main effect of practice, *F*(5,70) = 14.58, *p* < .001. A post-hoc Paired Samples t-tests indicated that the first block was significantly slower than all other blocks, and blocks 2 and 3 were significantly slower than block 6. There was no other significant difference between blocks.

### MT of the Posttest

**Fig 5:**
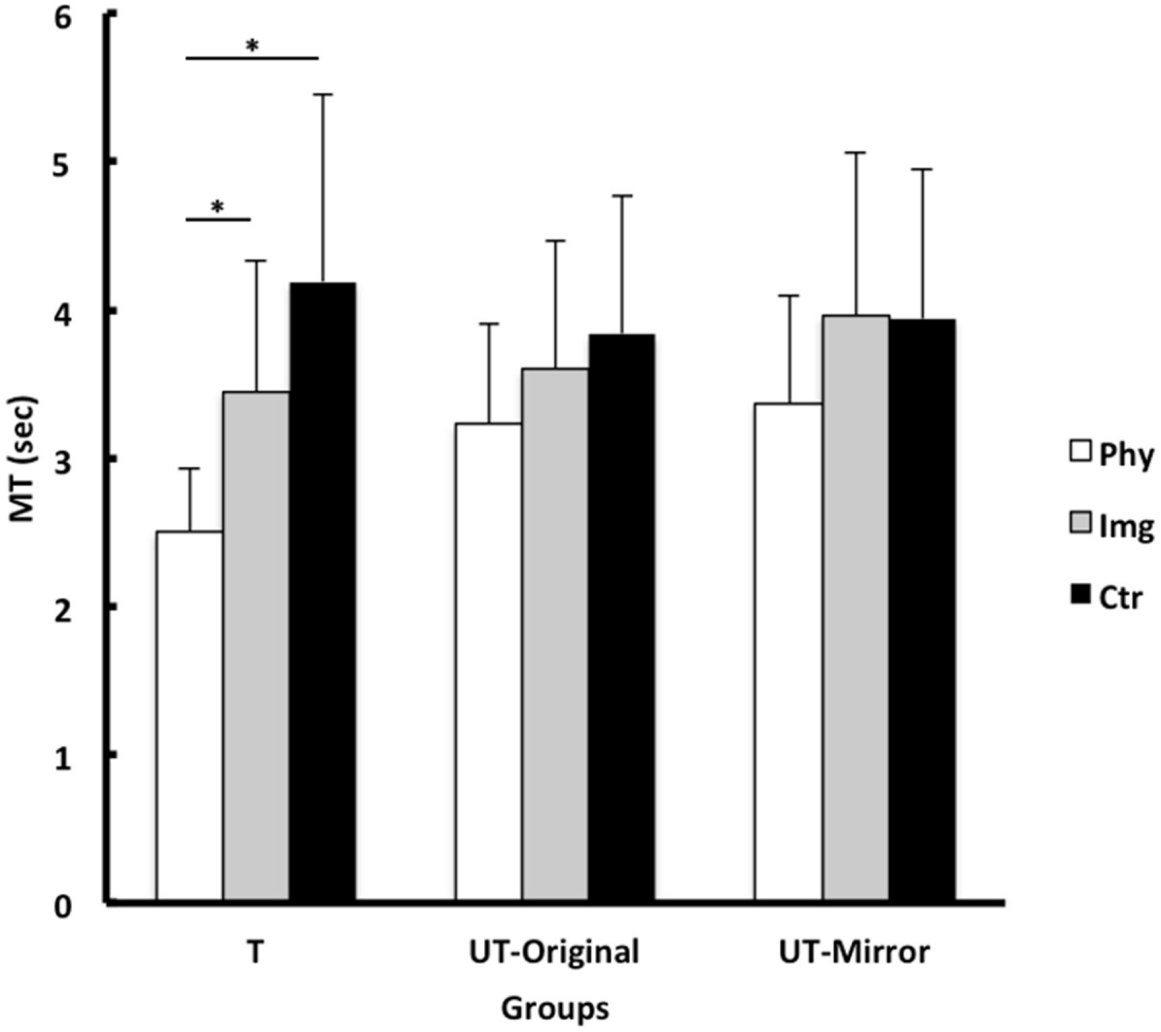
Posttest MT. Means and SDs of movement time (MT) of the posttest trials across each training condition for the trained hand test (T), untrained hand mirror ordered test (UT-Mirror), and untrained hand original order test (UT-Original) conditions in Experiment 2.

Fig 5 presents the means and SDs of MT for the posttest trials. The 3 x 3 (Group x Test Type) ANOVA with repeated measures on the second factor indicated significant main effects for both the Group and Test Type factor, *F*(2, 42) = 5.14, *p* < .05 and *F*(2, 84) = 7.78, *p* < .01, respectively. More importantly, the interaction between practice group and test type was significant, *F*(4, 84) = 7.71, *p*< .01.

To further explore the interaction effect, separate one-way ANOVAs were conducted for each of the three test types (i.e., trained-hand test, untrained-hand original-order test, and untrained-hand mirror-order test). The ANOVA for the trained-hand test indicated a significant group effect, *F*(2, 42) = 17.06, *p* < .001. Bonferroni post hoc tests indicated that the physical group (*M* = 2.50s, *SD* = .45) was significantly better than the imagery (*M* = 3.45s, *SD* = .88) and control groups (*M* = 4.19s, *SD* = 1.26). Furthermore, no significant differences emerged between the imagery and control groups. The one-way ANOVAs for the untrained-hand showed no significant effects for either original-order nor mirror-order tests, *F*(2, 42) = 2.01, *p* = .15, and *F*(2, 42) = 1.84, *p* = .17, respectively.

## Experiment 2: Discussion

Experiment 1 compared the influence of imagery practice versus physical practice with respect to an extended practice duration and task complexity on bilateral transfer from a non-dominant limb (i.e., left) to the dominant limb. Experiment 2 built off the findings of experiment 1 by examining the extent of transfer from the dominant limb (i.e., right) to the non-dominant limb.

The results from Experiment 2 are both surprising and unexpected. First, contrary to the results of Experiment 1, the imagery group did not significantly outperform the control group when performing the task with the trained limb. Furthermore, the physical group did not significantly outperform either the control group or imagery group in the two bilateral transfer tasks (i.e., original- and mirror-order transfers). In other words, the performance advantaged observed for the physical practice group while using the trained limb did not translate to a performance advantage when performing the task with the untrained limb. To this extent, there was no advantage with respect to bilateral transfer.

The absence of a significant improvement in learning the sequential tapping task for the imagery group compared to the control group may be due to the relative simplicity of the task itself. Using the dominant hand made the task even less challenging than in experiment 1, which in turn may have reduced the possibility to distinguish a difference in learning between the two groups. It should be noted that, compared to physical practice, the learning effect of imagery practice is generally small [22] and the low “challenge point” of the task may have reduced imagery’s learning effect further as predicted by the Challenge Point hypothesis [23]. In their hypothesis on practice conditions, Guadagnoli and Lee [23] suggested that the effectiveness of a given practice condition (e.g., imagery practice) is affected partly by the difficulty, or lack thereof, of the task (i.e., challenge point). According to this hypothesis, an appropriate (optimal) challenge point will enhance skill learning and any challenge points below or above this optimal threshold may be detrimental to skill learning. Thus, the simplicity of performing the sequential task with the dominant limb may have resulted in a low challenge point, and thus it is not surprising that the imagery group did not significantly outperform the control group.

Surprisingly, there was a lack of transfer from the trained limb to the untrained limb for the physical practice group. This may again be due to the characteristics of the task. The computer keyboard tapping task used in the current study can be considered more as a visuomotor skill due to its high spatial constraints. A previous study by Sainburg and Wang [20] indicated that visuomotor skills favored the transfer from the non-dominant to the dominant hand, and less so the other way around.

## General Discussion

The study by Amemiya, Ishizu [14] in learning a sequence-tapping task indicated a strong and significant advantage of imagery practice over physical practice when performed with the trained limb (i.e., non- dominant limb) and during bilateral transfer with the untrained limb (i.e., dominant limb). Amemiya, Ishizu [14] suggested that this advantage might be due to the interference caused by physical practice [16] and the complexity of the mirror-order transfer. While acknowledging the potential interference of physical practice coupled with the complexity of the task (e.g., mirror-order direction), we proposed that an extended practice period would eliminate and/or reduce the interference caused by physical practice. In addition, it was also assumed that an original-order transfer test would be superior to a mirror-order transfer test across the two training groups according to the findings of previous studies [18, 19]

The overall results of the current study indicate that extended practice will eliminate the advantage of imagery practice over physical practice as presented by Amemiya, Ishizu [14]. In both experiments 1 and 2, the two physical practice groups performed significantly better than the two imagery groups on the posttests with the trained hand. This result is not surprising because, as Driskell, Copper [9] stated, imagery practice has an advantage in improving cognitive understanding of the movement, and thus could facilitate the formation of the fundamental movement patterns, which is important during the early stages of learning. However, imagery is not able to provide direct knowledge of results or visual and tactile feedback, which is critical for the improvement of performance during the later stages of motor skill learning. Thus, imagery practice might be as effective as or superior to physical practice during the early stage of learning when the understanding of movement sequences is a major concern [24, 25]. However, with extended practice, such as the one used in the first experiment, imagery training may be less effective than physical practice in enhancing the performance of the trained hand [9].

With respect to bilateral transfer, the results were more mixed for the two experiments (i.e., transfer directions). While both physical and imagery practice groups had significantly better performance in the two transfer tests (e.g., mirror- and original-order transfers) than the control group in Experiment 1. However, when it was examined with the transfer from non-dominant hand to the dominant hand, both training groups failed to show a significant improvement in transfer compared to the control group in Experiment 2. This finding may be best accounted for by the work of Sainburg and Wang [20] who found that visuomotor skills, such as the one used in our current study, favors the transfer from the non-dominant hand to the dominant hand, whereas dynamical skill favors the transfer from the dominant hand to non-dominant hand. Specifically, Sainburg and Wang [20] attribute the asymmetrical bilateral transfer of learning (e.g., bilateral transfer of learning is direction sensitive) to the possible usage of different cognitive strategies for different tasks and different transfer directions. That is, although the controller for each limb has access to information learned during the training of the opposite limb, each controller uses this information differently, depending on its unique proficiency for controlling specific features of movement [20]. Krakauer, Ghilardi [26] also argue that bilateral transfer of visuomotor skill learning and dynamical skill learning may be subserved by two distinct neural processes. The observation of better transfer from the non-dominant hand to the dominant hand compared to the other way around, as in the current study, may be due to the fact that the dominant hand controller (i.e., sensory motor cortical areas) may have better access to the information obtained during the non-dominant hand training but not vice versa in the learning of a computer-key tapping task (i.e., visuomotor skill), the access hypothesis as argued by Krakauer, Ghilardi [26] and Sainburg and Wang [20]. This access hypothesis is indirectly supported by van Mier and Petersen [27] to show that performance of a sequential task with either a dominant or non-dominant hand activated same cortical areas. However, due to the limitation of the current study, it is unknown what caused differences in such access to the information obtained during either the non-dominant hand or dominant hand training from different limbs, which should be determined in future studies.

It should be noted that the strong practice advantage for the physical group over the imagery group was mainly seen on the trained limb but little if any on the untrained limb, or during bilateral transfers as it was shown in Fig 3. In other words, the imagery group showed similar performance between the trained and untrained limbs that suggests that whatever benefit that the imagery group gained from mental practice was equally available to both trained and untrained limbs. That is, the information (e.g., spatial sequence learning) gained during early stage of physical training and the whole stage of the imagery training is equally shared by both trained and untrained limbs (e.g., effector independence). However, the later learning (e.g., motor sequence learning) may be more effector-dependent as shown by the greater benefit for the trained limb than the untrained limb in the physical training group. On the basis of a series of studies using a sequence-learning task, Hikosaka, Nakahara [28] proposed that when sequential learning is associated with clear trial-and-error process, then it follows a gradual transition from a spatial sequence learning to a motor sequence learning. The former has strong demand in attention and the later does not have such demand. Their study [28] suggests that the spatial sequence learning enriches knowledge and/or understanding of sequential movement pattern and such knowledge/understanding could be used by any effectors (e.g., effector independence). In contrast, the motor sequence leaning enhances the coordination of muscle activations that could be effector dependent. In sum, the finding from the current study once again confirms that bilateral transfer occurs during the more cognitive or “algorithmic” phase of learning [21, 29].

In the present study, it was also predicted that differences would emerge between the training groups in regards to the extent of bilateral transfer when performing the sequential task in either a mirror order or original order movement sequence. This prediction was based on the assumption that imagery training leads to better transfer of skill on tasks that have a higher degree of complexity [9, 16]. In the case of the present study, it was assumed based on previous work [18, 19] that the mirror order sequential task would be cognitively more demanding, and thus better bilateral transfer performance would be associated with participants who trained via imagery. However, results did not support this prediction. As mentioned above, there was no evidence of a benefit to bilateral transfer for either the physical training group or the imagery training group in experiment 2. Thus, it is not surprising that a difference between the bilateral transfer tests were not found (e.g., mirror-order and original order tests) given that for visumotor skills transfer tends to be in the direction of non-dominant to dominant hand. However, more surprising was the lack of differences between bilateral performance in the mirror and original order in experiment 1. Specifically, no differences emerged between the physical practice and imagery practice group when performing either the original order or mirror ordered tests.

The predicted differences in performance between the physical and imagery group on the mirror and original order sequential task were based on the assumption that the mirror and original order task represent tasks of varying cognitive difficulty. However, the lack of findings may suggest that the mirror order and original order were not significantly different in terms of difficulty. It is possible that the extended practice with the trained hand led to substantial gains in performance when performing with the untrained limb (i.e., bilateral transfer). If it is the case that extended practice leads to even greater performance on the bilateral task, then the bilateral task regardless of mirror or original order may have been relatively easy for the participants given their extensive training on the task. To this extent, neither the original order nor the mirror order constituted a significantly different degree of difficulty. In order to test this assumption, future research should examine the performance differences between the mirror and original order task after only a brief training period, and again after an extended training period. Based on our account, differences between the orders may be more evident during early learning when the skill is less well learned (thus potentially less bilateral transfer), than later in practice.

In summary, the results of the current study indicate that, with a sustained practice period, physical practice is better than imagery practice in enhancing the performance on the trained hand and is equal to, if not better than, imagery practice in bilateral transfer. In addition, the current study provides further evidence of a positive transfer from non-dominant hand to dominant hand direction but not vice versa when sequential tapping tasks are used. These findings have clinical implications in terms of optimizing practice schedules. For example, the bilateral transfer of a learned skill is often one of the key concerns and/or goals in rehabilitation training with stroke patients. Largely, these individuals can only voluntarily move one side of their body. Practitioners, who are treating this hemispheric disability, often have stroke patients practice their intact, unaffected limbs physically and mentally with expectations or hope that this will facilitate rehabilitation of the limb of the affected side. Based on the current findings, a practitioner may consider applying imagery practice during the early stages of the rehabilitation and should apply more physical practice in the later stages of rehabilitation. As all other studies, the current study has some limitations. First, the current study did not examine brain activities and therefore, could not answer questions such as what are the factors that caused differences in access to the gained information by the dominant hand and non-dominant hand. Second, the results of learning and bilateral transfer in the imagery group are actually a combined effect of both imagery and observation because the subjects in this group watched video and conducted imagery during the practice. Thus it is unknown if 100% of imagery or observation would change any in the results. And the last but not the least, the current study did not conduct long-period either retention or transfer test (i.e., There were only 5 minutes gap between acquisition phase and the transfer test phase.) Thus, the current study could not predict if the benefit shown by the physical group over the imagery group would be sustained or be a long-period effect. Therefore, future study or studies are needed to address the limitations in the current study.
